# Supplementary material for: The pupillary light response as a physiological index of aphantasia, sensory and phenomenological imagery strength
Source: eLife. 2022 Mar 31;11:e72484. doi: 10.7554/eLife.72484 (PMC9018072; doi:10.7554/eLife.72484)
Supplement: Figure 1—source code 1. — Source code file 1 provides the r code for the LME used to analyse the vividness data in Figure 1D. [file elife-72484-fig1-code1.zip › LME_r_code_pupil_vividness.rtf]

R code for mixed linear models - Written by Rebecca Keogh - lme4 packagelibrary (lme4)setwd (“data.csv”) % Sets path - use cd of where csv is%% Can also do this by going to misc and changing by handdata = read.csv("data.csv”)  % Loads data which(!complete.cases(data)) % Shows which have missing dataVIVIDNESS%%make variable categoricaldata$Vivid_Rating = factor(c(data$Vivid_Rating))%%checkdata$Vivid_Rating%% Assumptionsplot(fitted(tdcs.model),residuals(tdcs.model)) % Linearity want to see random spatter of blobs dots - also homoscadaictyhist(residuals(tdcs.model)) - normality qqnorm(residuals(tdcs.model)) - normality - should form diagonal line%% Only intercept not slopevivid.model = lmer(Pup_dil ~ Vivid_Rating + SS + (1|ID) ,data=data, REML=FALSE) % Runs LME on data - subjects is random effect with intercept + slope controlled for - rest are fixedsummary(tdcs.model) % Prints model variables etcnull.vivid.model = lmer(Pup_dil ~  SS + (1|ID) ,+ data=data, REML=FALSE) % Runs LME on null model - subjects is random effect with intercept + slope controlled for - rest are fixedsummary(null.vivid.model) % Prints model variables etcAnova - compares modelsanova(vivid.model,null.vivid.model)
