# Supplementary material for: The pupillary light response as a physiological index of aphantasia, sensory and phenomenological imagery strength
Source: eLife. 2022 Mar 31;11:e72484. doi: 10.7554/eLife.72484 (PMC9018072; doi:10.7554/eLife.72484)
Supplement: Supplementary file 1. — This file provides the fixed effects estimates for the LME run on the pupil-difference scores for the general population as a function of vividness ratings and set size. [file elife-72484-supp1.docx]

**Supplementary File 1**

| Fixed effects: Vividness Model (Pupil_dilation ~ Vivid_Rating + SetSize + (1 \| ID)) |
| --- |
| Estimate Std. Error t value |
| (Intercept) 0.15909 0.03645 4.365 |
| Vivid_Rating2 0.07556 0.02563 2.948 |
| Vivid_Rating3 0.17058 0.02488 6.855 |
| Vivid_Rating4 0.14879 0.02528 5.887 |
| SS -0.07980 0.01663 -4.797 |

**Supplementary File 1:** Fixed effects for LME of vividness data (see Figure 1D)
